# Special Issue on “Advances in Socio-Economic Research on Ageing”

**DOI:** 10.3390/ijerph18126337

**Published:** 2021-06-11

**Authors:** Cristina Gagliardi, Giovanni Lamura

**Affiliations:** IRCCS INRCA, Centre for Socio-Economic Research on Ageing, 60124 Ancona, Italy; g.lamura@inrca.it

This Special Issue provides the readers of the *International Journal of Environmental Research and Public Health* a multidimensional overview of recent developments in the field of socio-economic gerontological research. It does so by recognising, in the first place, that population ageing is no longer a phenomenon only characterising wealthier countries, but rather a trend concerning an increasing number of middle-income and even low-income nations. This is well reflected by the number of contributions coming from researchers reporting about the experience of Asian countries in a series of ageing-related issues, which provide an interesting and enriching integration of the—traditionally more numerous—studies focussing on European and Northern American contexts. The perspective offered by this mix of countries contributes to gaining a better and more comprehensive understanding of the partly culturally sensitive and multifaceted process of ageing; and of the demographic, social, economic and political challenges it poses, both at a micro and macro level. The works collected under the umbrella of this Special Issue range from the broad and encompassing topic of active ageing—a concept which is gaining an increasing interest across the globe [1], and often in connection with that of healthy ageing [2,3] to specific (e.g., physical and cognitive) sub-dimensions of health deterioration in later life and up to formal and informal components of long-term care. While they cannot certainly represent an exhaustive overview of the many developments occurring in parallel in the mentioned areas, these contributions provide a glimpse into recent scientific progresses in the above mentioned fields, both by using the means of single-country and comparative cross-national studies.

Active Ageing

The largest group of works included in this collection concerns the broad topic of active ageing in its different facets. This concept is defined by the European Commission as “helping people stay in charge of their own lives for as long as possible as they age and, where possible, to contribute to the economy and society” [4], As such, the concept refers to phenomenon which takes place in different life domains, such as the work place, within civil society, and along one’s own life. The concept of active ageing thus requires, in order to be adequately addressed, a cross-sectoral approach to analyse and support the needs of an ageing population. In this regard, one of the perspectives that has been widely debated in recent years, especially within the European context, has been that of “extending working life”. This is a matter that embraces both macro-level concerns—such as for instance those pressing for a postponement of retirement age as a means to improve the financial sustainability of pension systems—and micro-level aspects, such as those dealing with the implementation of practices and interventions to guarantee the health and safety of older workers.

Within the vast topic of active ageing, two of the presented articles focus on specific aspects of employment in later life. The paper by Andrea Principi and colleagues concerns the management of ageing workers by highlighting the different human resource policies at different companies aimed at lengthening the working lives of older employees [5]. Using data from a large European study involving companies from six countries, three types of approaches are identified: the prevention of early retirement; the delay of retirement; and the recruitment of employees who are already retired. These approaches are compared with the companies’ main drivers to improve the performance of older workers and their working conditions or to reduce costs. The main findings emerging from this analysis underline the importance of promoting organizational practices to improve the employability and working conditions of older workers by using innovative tools, such as laws contemplated in agreement with social partners to fund initiatives extending working life by means of a stronger appreciation of the qualities and skills characterising older workers. A second paper, by Tianan Yang et al. [6], assesses factors of work environment that may facilitate access to job resources in a sample of American ageing workers. This is considered an important aspect as it may improve one’s sense of control (measured in relation to personal mastery and perceived constraints) in relation to presenteeism (defined as a potential productivity loss attributable to health problems or other events that adversely affect employees). The inverse association found by authors between personal mastery and presenteeism leads them to conclude that, in order to promote confidence and a pro-active approach among ageing workers, managers should reduce pressure on them, promote fairness and improve the work environment as part of a broader management strategy aimed at increasing enthusiasm among employees and thereby augmenting their productivity.

Another important aspect of active aging is the practise of volunteering as a form of social engagement and participation. Social engagement in later life has been long recognized as a protective factor in terms of health status, cognitive functioning and mortality. Selfless motives such as helping others and providing support have been shown to be directly related to better physical and mental health. Two papers of this Special Issue address this specific topic from different perspectives. The first one, by Marco Socci and colleagues [7], begins with the acknowledgement that older individuals may act as key players of social change in local communities by leveraging social entrepreneurship as an important tool to tackle unmet social challenges, driving social change and driving social innovation; and, as such, to alleviate social problems, catalyse social transformation and create and sustain social value. The role of older individuals acting as senior social entrepreneurs by engaging in volunteering activities in five European countries was examined in this work. While highlighting the potential of this approach to create high social value with a relatively low economic outflow, scarcity of funds still remains a major challenge such that policy efforts to guarantee sufficient funds in this regard represent a necessity. The perspective of the pilot study carried out by Cristina Gagliardi et al. [8] is a different one, as it investigates the feasibility of an environmental volunteering program involving park restoration and social activities for older people in Italy. By means of qualitative data collected at two different time points, this piece of research highlights that this kind of activity can significantly improve participants’ level of physical activity, subjective life satisfaction, identity and positive feelings at the follow-up—thanks to a pro-environmental attitude of the involved volunteers.

A third and final component addressed by contributions included in this Special Issue concerns policy-related aspects of active ageing. This is conducted, however, with very different methods by the two articles focussing on this dimension. The first, by Roberto Falanga and colleagues [9], investigates how civic participation by senior citizens in policy-making may contribute to developing policies and design services that respond to the needs of older people and are relevant to their circumstances. By means of a review of the literature, a consultation of national policy experts and the analysis of exemplary case studies in the European Union and associated member states, this work identifies four main patterns through which older individuals are able to act at an institutional level, adopting consultative or co-decisional participatory approaches in policy design or policy implementation. These patterns are represented to varying degrees at different geographical levels (national, regional and local) with different actor configurations (appointed, elected/nominated and corporate representation) and varying degrees of institutionalisation (temporary/permanent), while case studies illustrate approaches taken to enhance the quality and effectiveness of public services for senior citizens. While additional research will be required to cast further light on the conditions facilitating the civic participation of senior citizens from this perspective, the second article addressing this topic—by Francesco Barbabella et al. [10]—is represented by an empirical case study, based on the ongoing Plan-of-Action adopted by the Italian government to promote active ageing in this country. The core message emerging from this study underlines the need for systematic and coordinated stimuli at different governance levels (regional as well as national) to achieve a stronger promotion of active ageing across the country. This article analyses how, starting from the common principles and (both national and international) good practices in the field of active ageing, the first national Plan-of-Action has been co-designed for the period 2019–2022 to cover the traditional policy cycle, including the stages of agenda setting, policy formulation, decision-making, implementation and monitoring. The authors highlight the necessity of stimulating connections between public, non-profit and research stakeholders at different levels in order to increase cross-sectoral awareness among different policy areas linked to social participation and inclusion in old age following a participatory approach.

Selected Risks of Health Deterioration in Later Life

A second group of five articles included in this Special Issue focusses on specific widespread risks of health (deterioration) in later life by taking into account the situation in some of the most populous Asian countries. Maintaining health and quality of life are among the main challenges of ageing societies worldwide, and understanding why some population subgroups are more subject than others to illnesses and/or poor wellbeing is of highest importance. Marcus Yu Lung Chiu and colleagues [11] tackle this issue by putting under investigation distressing urinary problems of newly retired men in Hong Kong and testing their associations with mental health, the self-stigma of seeking help, fatigue, self-efficacy and self-esteem. Their result highlight that those who reported these symptoms showed significantly poorer mental health, reported more fatigue symptoms, were less satisfied with their sexual relationships and overall self-esteem and were less able to stop unpleasant thoughts or to get social support than the non-distressed group. Gender-specific cultural perceptions of masculinity and decreased sexual vigour might have affected participants’ willingness to seek help at an early stage. Targeted health education, mutual support groups and sensitively designed services at the community level are suggested as possible strategies to effectively address these physical and mental health issues.

Another important health-related issue affecting many older people is the fear of falling. This is a syndrome that potentially leads to the avoidance of activities related to daily living in older people and Long Hoang Nguyen et al. [12] investigates this phenomenon, which represents a major cause and consequence of falls and associated factors, in specific settings, in association with health-related quality of life in older patients hospitalised due to fall-injuries in seven hospitals in Vietnam. Results indicate a substantial prevalence of fear of falling among older patients admitted to the hospital after falls, with a substantial negative impact on the patient’s quality of life. These findings suggest the need for interventions aimed at improving knowledge and providing counselling and guidelines about fall prevention in patients and caregivers in order to reduce the overall burden of falls in older people.

The increasing longevity of large shares of the population is strongly associated with the growing number of older people reporting a deterioration of cognitive abilities and empirically sound knowledge in this area is of great importance to improve perspectives for ageing well in the future. The longitudinal study of Fan Yang and colleagues [13] addresses this issue by examining the trend in cognitive functions among different socio-economic cohorts of older adults of different genders and areas of residence, using data of the Chinese Longitudinal Healthy Longevity Survey (CLHLS). They found a greater increase in cognitive functions per birth cohort among older Chinese adults with less education, lower household income and who were economically dependent. Furthermore, the magnitude of differences in cognitive function between older adults with higher and lower incomes was larger among those living in rural areas than among those living in urban areas. The authors underline that rural residents with lower income levels may have lower resources and opportunities for social engagement and interaction to prevent their cognitive decline than their counterparts, concluding that effective public intervention targeting socio-economically disadvantaged populations is still necessary.

Starting from the observation that ageing poses increasing challenges in India, including a series of gender specific issues due to the disadvantaged status of older women in this country, the work of Ildikò Asztalos Morell et al. [14] explores the health and well-being of older women that lacking independent income in this nation, by focussing on their agency freedoms. By the means of four life stories, different patterns illustrate how older Indian women position themselves in different types of extended families—an institution that has traditionally been and still is the main normative and, by law, sanctioned source of care for frail older persons in India—and the impact that the different positioning may have on their health and quality of life.

Finally, in global pandemic times as the current one, it is not surprising that this Special Issue also includes an article addressing the impact of COVID-19 related restrictions on the mental and physical health of older people by analysing how new communication technologies have become essential to maintain their social contacts during the “lockdown” period imposed to prevent the contagion. The study by Elena Rolandi and colleagues [15] does so by exploring how older adults who had previously been trained in the use of Social Networking Sites (SNSs)—the technical term employed to refer to internet-based social media platforms to stay connected with friends, family or peers, such as Facebook, Instagram or Twitter—have experienced their use during the lockdown period. Their findings support the utility of training older adults for SNSs use in order to improve their social inclusion, even in extreme conditions of self-isolation and perceived vulnerability and they highlight that those participants trained for SNSs use reported significantly higher usage of SNSs and reduced feelings of being left out, despite a lighter reduction in social contacts.

Long-term Care

The last, but not the least relevant, group of papers—in an ideal line of reasoning moving from an active and independent life stage to a frail and more dependent period of the life span—deals with the topic of long-term care. This is a global challenge concerning the need to ensure adequate measures to meet health-related care needs due to a chronic illness or disability—which are to a large extent (but not exclusively) age-related—in order to ensure a good quality of life when people develop frailty and lose the ability to interact independently with others and the surrounding environment. While long-term care has become politically relevant, due to the costs associated with the care of a growing number of frail older people (for recent projections on expenditures in this area within the European Union cf. the just released Ageing Report by the European Commission 2021 [16]), three papers focus on the crucial, but still too often-neglected or only partly considered phenomena of informal care, social innovations and migrant care work.

As for the first topic, there is no doubt that the overwhelming majority of older people who need help in performing basic everyday activities rely on the assistance of informal caregivers, such as family members or friends providing unpaid help. Estimating the number of informal caregivers in ageing societies is crucial for planning long-term care measures and policies. Starting from this premise, the study of Aviad Tur-Sinai et al. [17] used the European Health Interview Survey (EHIS), the European Quality of Life Survey (EQLS) and the Study on Health and Ageing in Europe (SHARE) to measure the prevalence of informal caregivers in the European population. Their findings not only reveal common trends across the three surveys but also reveal a series of disparities, including, for several countries, a large variance among the three sources of data with regard to the average share of informal caregivers. The authors conclude that solutions are needed to overcome this uncertainty in order to enable policy makers across countries to count on a more harmonized body of reliable data on such a fundamental component of care systems; if evidence-based, targeted policies and interventions in this field are to be adopted.

Moving on to the second topic, Georgia Casanova and colleagues [18] illustrate which social innovations have been recently implemented in Italy by means of a comparative approach underpinned by the findings of two European projects. While formulating a series of suggestions on how social innovation can be promoted by strengthening the integration and coordination of available services and resources, the main conclusion of this piece of research is that building new collaborations between different stakeholders, via multi-stakeholder networks, appears to be the prevalent strategy to promote social innovations in the long-term care sector of this country.

Last but not least, in the background of the observation that long-term care provision to community dwelling frail older adults in many countries is partly provided by live-in migrant care workers, the study of Oliver Fisher et al. [19] provides a scoping review of recent contributions in this field with regard to Italy and Israel; the two of the countries in which this phenomenon is most widespread. The novelty of this piece of research lies in the fact that, while there have been many studies that detail the labour rights violations experienced by migrant care workers, this is the first review that develops themes around the underlying causes of these violations. By thematically analysing the findings of recent studies and current gaps in existing knowledge, this scoping review shows that migrant care workers in both Italy and Israel face many of the same challenges in accessing decent work opportunities despite contrasting employment and migration policies in each country. Thus, hinting that some structural issues may well benefit from an international approach aimed at promoting stronger attention and common monitoring tools on this often-neglected topic.
